# Defect Passivation in Perovskite Solar Cells Using Polysuccinimide-Based Green Polymer Additives

**DOI:** 10.3390/polym17050653

**Published:** 2025-02-28

**Authors:** Sergey S. Kozlov, Olga V. Alexeeva, Anna B. Nikolskaia, Vasilisa I. Petrova, Olga K. Karyagina, Alexey L. Iordanskii, Liudmila L. Larina, Oleg I. Shevaleevskiy

**Affiliations:** 1 Emanuel Institute of Biochemical Physics, Russian Academy of Sciences, 119334 Moscow, Russia; sergeykozlov1@gmail.com (S.S.K.); anickolskaya@mail.ru (A.B.N.); balagur.sh@yandex.ru (V.I.P.); olgakar07@mail.ru (O.K.K.); llarina@yahoo.com (L.L.L.); shevale2006@yahoo.com (O.I.S.); 2 N.N. Semenov Federal Research Center for Chemical Physics, Russian Academy of Sciences, 119991 Moscow, Russia; aljordan08@gmail.com

**Keywords:** perovskite solar cells, green polymers, perovskite modification, defects passivation, efficiency, stability

## Abstract

Controlling traps and structural defects in perovskite absorber layers is crucial for enhancing both the device efficiency and long-term stability of perovskite solar cells (PSCs). Here we demonstrate the modification of perovskite films by introducing low-cost green polymers, polysuccinimide (PSI) and polyasparagine (PASP), into the perovskite layer. Structural, morphological and optoelectronic properties of polymer-modified perovskite films were probed by scanning electron microscopy (SEM), X-ray diffraction (XRD), Fourier transform infrared (FTIR) and UV-Vis spectroscopy. The incorporation of PSI triggers interactions between the polymer and perovskite, leading to the passivation of surface defects at the grain boundaries and improved morphology of perovskite films. This defect passivation boosted PSC performance, providing power conversion efficiency (PCE) values up to 20.1%. An optimal polymer concentration of 0.1 mg/mL in the perovskite precursor solution was identified for an improvement in the photovoltaic performance. It was shown that the primary factor leading to the observed enhancement in the power conversion efficiency for PSI-modified PSCs is the increase in the lifetime of charge carriers due to the efficient passivation of surface defects and suppression of recombination losses. Additionally, PSI-modified PSCs demonstrated enhanced stability, retaining over 80% of their initial efficiency after 40 days of storage under ambient conditions without encapsulation. The obtained results highlight the effectiveness of green polymer additives in passivating surface defects in perovskite films and provide a viable approach for improving the stability and performance of perovskite solar cells.

## 1. Introduction

Hybrid organic–inorganic perovskites have attracted ever-increasing attention as promising materials for photovoltaic applications due to their exceptional optoelectronic characteristics, including strong light absorption, tolerance to defects and long charge carrier diffusion length [1,2,3]. Together with the possibility of low-cost solution processing, this has brought about the development of perovskite solar cells (PSCs), a new class of photovoltaic devices [4,5,6]. Over the last decade, the power conversion efficiency (PCE) of PSCs has been boosted from 3.8% to 26.0%, making them competitive with conventional solar cell technologies based on crystalline silicon [7,8]. Solution-processed perovskite films inevitably possess different types of intrinsic defects in the bulk or at the grain boundaries, like vacancies, undercoordinated ions and interstitials [9,10]. The formation of defects in perovskite films is detrimental to the performance and stability of PSCs, since they act as non-radiative recombination centers and degradation sites. Therefore, the control of defects in the perovskite films is essential for enhancing the efficiency and long-term stability of PSCs [11].

The issues related with perovskite film quality and defect formation were addressed using various approaches, including the introduction of intermediate passivation layers [12], tailoring perovskite crystallinity kinetics [13,14] and additive engineering [15,16]. The latter represents an effective strategy to control perovskite crystallization and mitigate the unwanted defects in the perovskite structure. It is well established that small molecules with an electron donor group (Lewis base) could improve perovskite crystallization and provide passivation of the halide vacancy defects through coordination with undercoordinated Pb_2_^+^ ions [17]. Small molecule passivators have some disadvantages, namely, high volatility, mobility and a limited number of functional groups. Therefore, long-chain polymers with Lewis base functional groups have received intensive attention as additives for perovskite materials [18,19]. Recently, different synthetic polymers have been used to stabilize the perovskite structure, passivate surface defects and increase the moisture and thermal stability of perovskite films: PMMA [20], poly(ethylene imide) [21], polyvinylpyrrolidone (PVP) [22] and poly(propylene carbonate) (PPC) [23]. The introduction of polymers into the perovskite material affects the structural and optoelectronic characteristics of perovskites, promoting enlarged grain size, uniform morphology and decreased defect density, thus leading to the improved performance and stability of PSCs [24,25,26,27].

However, polymer additive engineering of perovskite materials has particular issues that should be resolved in order to achieve cost-effective and sustainable PSC fabrication. It was previously shown that relatively large amounts of polymer additives (from tenths up to few wt.%) are required to enhance the optoelectronic characteristics of perovskite films and increase the photovoltaic performance of PSCs [19,28]. High levels of polymer additives could lead to the formation of unwanted polymer phases inside the perovskite layer or impede charge transfer due to the electrically insulating nature of the polymer [22]. Moreover, the majority of polymers used as additives for perovskites are synthetic ones and only a few reports consider the application of “green polymers” or biopolymers in the additive engineering of perovskite materials [29,30,31]. Therefore, it is of great significance to develop novel cost-efficient green polymer additives that could enhance both the photovoltaic performance and stability of PSCs, providing those effects at minimal possible concentrations.

Polysuccinimide, a non-toxic and cheap green polymer obtained by thermal polycondensation of L-aspartic acid, is considered as a promising substitute of the synthetic polymers in various applications [32,33,34]. The high molecular dipole moment of succinimide and the presence of carbonyl groups in PSI promote molecular interactions with the perovskite precursors, i.e., CH_3_NH_3_^+^ cations and iodoplumbate anions. Additionally, PSI is a thermostable polymer and has hydrophobic properties, which could help to induce the moisture resistance of perovskite films. PSI derivatives, such as polyaspartic acid (PASP), could also be potentially effective in the defect passivation of perovskites due to the presence of carboxyl and carbonyl functional groups. Recently, succinimide was incorporated as an additive in the perovskite active layer, which led to the efficient passivation of undercoordinated Pb^2+^ defects and provided improved PSC performance and stability [35]. However, the application of PSI as a polymer additive in hybrid perovskites has not been reported yet.

In this work, we demonstrate the facile modification of perovskite films by the incorporation of polysuccinimide (PSI) and polyasparagine (PASP) green polymers into the perovskite layer. Structural, morphological and optoelectronic properties of polymer-modified perovskites were probed by FTIR, UV-Vis spectroscopy, time-resolved photoluminescence (TRPL), SEM and XRD. Our findings demonstrate that a small polymer concentration (0.1 mg/mL in the perovskite precursor solution) was optimal for perovskite modification and an improvement in the morphology and optoelectronic properties of perovskite films. This consequently leads to increased PCE values up to 20% for planar n-i-p perovskite solar cells based on PSI-modified perovskite layers. The key factor for the observed boost in the performance of PSI-modified PSCs was the suppression of recombination losses and the increase in the lifetime of charge carriers due to the efficient passivation of surface defects. The obtained results provide a promising approach for efficient application of low-cost green polymer materials in perovskite photovoltaics.

## 2. Materials and Methods

### 2.1. Materials

The tin (IV) oxide colloid precursor solution (15% in H_2_O) was obtained from Alfa Aesar (Thermo Fisher GmbH, Kandel, Germany). Methylammonium iodide (MAI) and lead iodide (PbI_2_) were purchased from TCI (Tokyo Chemical Industry Co., Tokio, Japan). Fluorine-doped tin oxide (FTO)-coated glass substrates (8 Ω∙cm^−2^), diethyl ether (DE), chlorobenzene (CB), N,N-dimethylformamide (DMF), N-Methyl-2-pyrrolidone (NMP), titanium diisopropoxide bis(acetylacetonate) solution (75 wt.% in isopropanol), Spiro-MeOTAD, bis(trifluoromethylsulfonyl) imide lithium salt (Li-TFSI) and FK 209 Co(III) TFSI salt were purchased from Sigma-Aldrich (St. Louis, MO, USA). All materials were used as received, without further modifications.

### 2.2. Polymer Synthesis

**Figure 1:**
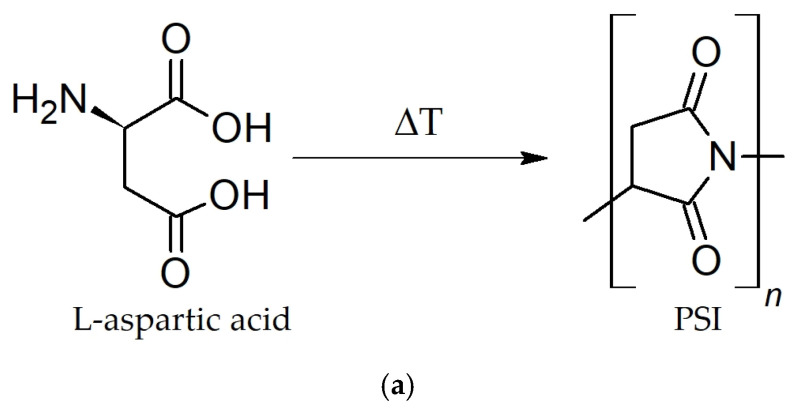

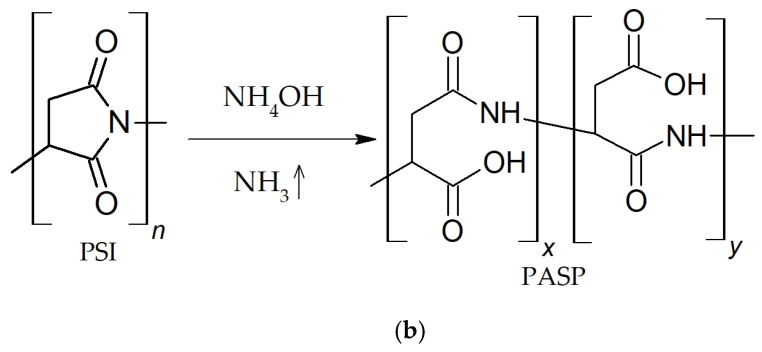
Schematic representation of PSI (**a**) and PASP (**b**) synthesis. Thermal polycondensation of L-aspartic acid was performed at 240 °C for 2.5 h.

Polysuccinimide (PSI) was synthesized via solid-state thermal polycondensation of L-aspartic acid, as described previously [36]. Briefly, L-aspartic acid (Sigma-Aldrich, St. Louis, MO, USA) was grinded in a mortar and heated at 240 °C for 2.5 h in order to achieve a high conversion degree during polycondensation (Figure 1) and an average M_w_ of 14,000 Da [37]. No further treatment was used for the synthesized PSI powder. The structure of synthesized PSI was confirmed by nuclear magnetic resonance (NMR) analysis using a Brucker Avance III 500 spectrometer (Bruker Corporation, Billerica, MA, USA). Nuclear magnetic resonance (NMR) studies were carried out in DMSO-d_6_.

As was shown previously, PSI could be easily hydrolyzed in alkaline conditions with the formation of polyaspartic acid (PASP) [33]. PASP was synthesized by dissolving the obtained PSI powder in NH_4_OH aqueous solution, with constant stirring for 1 h at 25 °C. Anhydrous ethanol was further added to the polymer solution and it was thoroughly stirred until the precipitate was formed. The precipitate was further filtered and dried in an oven at 40 °C for 12 h to obtain PASP.

The perovskite precursor solution was prepared by dissolving 1.5 mM of PbI_2_ and MAI in the mixed DMF/NMP solvent (4:1 *v*/*v*), with the addition of 2% *v*/*v* of deionized water [38]. Synthesized PSI and и PASP powders were dissolved in DMF at 5 mg/mL concentration. These stock solutions were added in various amounts to the perovskite precursor solution to obtain the total polymer concentration of 0.1 mg/mL, 0.5 mg/mL and 3.0 mg/mL. In order to obtain adduct powders, the perovskite precursor solutions with (0.1 mg/mL concentration) or without the polymers were added dropwise into 10 mL of diethyl ether. After stirring the solution for 10 min, the precipitates were recovered, washed with diethyl ether and dried under vacuum.

### 2.3. Device Fabrication

The FTO–glass substrates were consecutively cleaned with Triton X-100, ethanol and acetone in an ultrasonic bath for 15 min. The TiO_2_ compact layer (cTiO_2_) was prepared by spin-coating of 0.15 M titanium diisopropoxide bis(acetylacetonate) solution in 1-butanol on the FTO–glass substrate at 3000 rpm for 30 s, with subsequent drying at 500 °C for 30 min. Subsequently, the SnO_2_ thin film was spin-coated onto the FTO/cTiO_2_ substrate using a diluted tin oxide colloid precursor (2.67%, diluted by deionized water) at 3000 rpm for 30 s and was annealed in the ambient atmosphere at 150 °C for 30 min to obtain the electron transport layer (ETL). Next, the cooled down ETL was additionally treated with UV-ozone for 15 min. Perovskite layer deposition was carried out under ambient conditions (~30% humidity). The perovskite precursor solution was spin-coated on the FTO/cTiO_2_/SnO_2_ substrate at 4000 rpm for 25 s and 0.3 mL of diethyl ether was dripped on the rotating substrate at the 10th second as an antisolvent. The obtained perovskite layer was annealed at 65 °C for 5 min and 120 °C for 10 min. A hole-transporting layer (HTL) was spin-coated on top of the perovskite layer at 3000 rpm for 30 s using a solution consisting of 72.3 mg Spiro-OMeTAD, 28.8 μL 4-tert-butylpiridine, 17.5 μL bis(trifluoromethane) sulfonimide lithium salt (Li-TFSI) solution (520 mg Li-TSFI in 1 mL of acetonitrile) and 10 μL FK 209 Co(III) TFSI solution (300 mg FK 209 Co(III) TFSI in 1 mL of acetonitrile) in 1 mL of chlorobenzene. Finally, an 80 nm Au layer was deposited as the top contact using thermal evaporation.

### 2.4. Characterization

XRD patterns of the as-prepared perovskite films were acquired using a DRON-3M X-ray diffractometer (Burevestnik, Saint Petersburg, Russia) with Cu-Kα radiation (λ = 1.5405 Å) as the X-ray source. Scans were taken in the 2θ range of 10–70° with a 0.05° scan step. SEM images of perovskite films prepared on FTO–glass substrates were obtained by a Hitachi SU8000 field-emission scanning electron microscope (Hitachi High-Tech, Tokyo, Japan) equipped with the Oxford Instruments X-max EDX system (Oxford Instruments, Abingdon, UK). The sizes of the perovskite crystalline grains were obtained from SEM images using the ImageJ software (version 1.54k). Atomic force microscopy (AFM) measurements were carried out on an NTEGRA Prima atomic force microscope (NT-MDT, Moscow, Russia) in tapping mode using NSG03 probes (resonance frequency 90 kHz, spring constant 1.74 N/m). UV-Vis absorption spectra of perovskite films on glass substrates were obtained using a Shimadzu UV-3600 spectrophotometer (Shimadzu Corporation, Tokyo, Japan) in the wavelength range of 350–850 nm. Steady-state photoluminescence (PL) was measured using the Ocean Optics Maya 2000 Pro fiber spectrometer (Ocean Insight, Saint-Petersburg, Russia). A semiconductor pulsed diode laser PicoQuant LDH-C 400 (PicoQuant, Berlin, Germany, 405 nm wavelength, 5 mW average power, 75 ps pulse width, 100 kHz pulse repetition rate) was used as the excitation source. Time-resolved photoluminescence (TRPL) measurements were performed using the PicoQuant PMA-C 192-N-M photodetector connected to the PicoQuant TimeHarp 100 correlator (PicoQuant, Berlin, Germany).

FTIR spectra of perovskite films were recorded using the Perkin–Elmer Spectrum Two FTIR spectrometer (PerkinElmer, Shelton, CT, USA) with a modified DRIFT (Diffuse Reflectance Infrared Fourier Transform Spectroscopy) attachment, allowing the measurement of solid samples [39,40]. Perovskite films were deposited on the thin Si substrates as described above. A small 13 mm diameter stainless steel table acting as a mirror was used and the infrared ray penetrated the sample placed directly on the table surface. The position of the sample holder was optimized prior to measurements using a clean metal roller. FTIR spectra were recorded in the range of 4000–400 cm^−1^ with a 4 cm^−1^ resolution using the average of 16 successive scans. In addition, FTIR spectra were recorded for the synthesized PSI and perovskite adduct powders to identify interactions between the polymer and the perovskite precursor. PSI and perovskite adduct powders with PSI and PASP were analyzed using a Bruker Tensor 27 IR Fourier spectrometer (Bruker corporation, Billerica, MA, USA) with the PIKE MIRacle™ accessory (PIKE Technologies, Madison, WI, USA) equipped with a germanium (Ge) crystal and an ATR attachment with a Teflon cell and cesium antimony electrode, which allows the measurement of solid samples. The powder sample was placed on the surface of the crystal and tightly clamped to ensure optical contact. IR spectra were recorded in the range of 4000–400 cm^−1^ with a resolution of 4 cm^−1^. Raman spectra of perovskite films were acquired using the NS240 Raman spectrometer (Nanoscope Systems Inc., Daejeon, Republic of Korea) with 532 nm excitation at the range of 2000–100 cm^−1^ and resolution of 4 cm^−1^.

The current density–voltage (J-V) characteristics of PSCs were measured under standard AM1.5G illumination using the Keithley SCS-4200 Semiconductor Characterization System (Keithley instruments, Solon, OH, USA) and Abet Technologies 10500 solar simulator with Xenon lamp (Abet Technologies, Milford, CT, USA) as a light source. The active area of PSCs, provided by the metal aperture mask, was 0.08 cm^2^. The average values of PV parameters (J_SC_, V_OC_, FF) for different PSC types were obtained for a series of 10 PSC samples. The external quantum efficiency (EQE) spectra were recorded using the QEX10 Solar Cell Quantum Efficiency Measurement System (PV Measurements, Point Roberts, WA, USA) in the wavelength range of 300–850 nm.

## 3. Results

### 3.1. PSI Synthesis and Characterization

**Figure 2:**
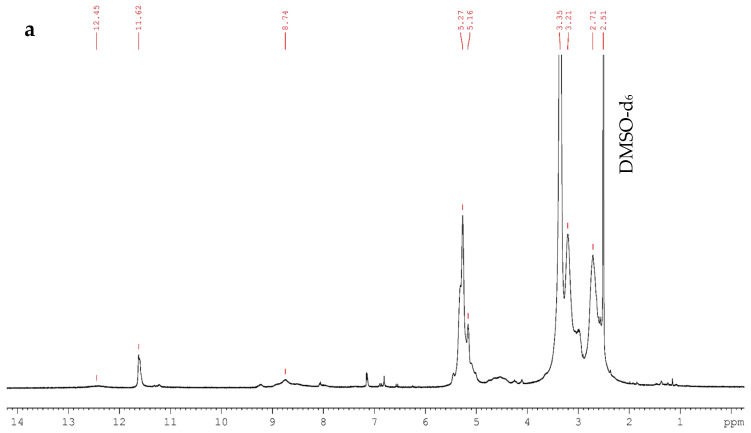

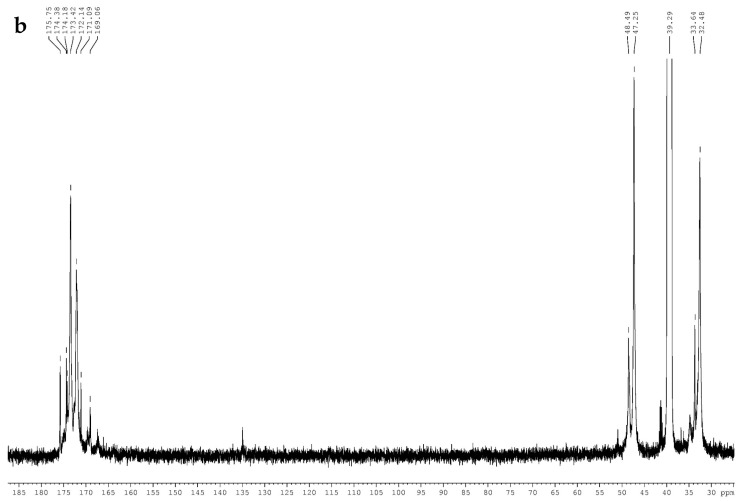
^1^H NMR (**a**) and ^13^C NMR (**b**) spectra of PSI in DMSO-d_6_ (^1^H frequency—500 MHz, ^13^C frequency—125 MHz, temperature—298 K).

Polysuccinimide (PSI) was synthesized by solid-state thermal polycondensation of L-aspartic acid under ambient conditions without the use of other chemicals; therefore, it could be regarded as green polymer [41,42]. The process is a two-stage process: In the first stage (around 1 h), PSI molecules with a molecular weight (M_w_) of around 10,000 Da are formed. During the remaining reaction time, the average M_w_ of the obtained PSI monotonically increases up to 20,000 Da [37,43]. The synthesized polysuccinimide was characterized by NMR spectroscopy in DMSO-d_6_ and the corresponding ^1^H NMR and ^13^C NMR spectra are presented in Figure 2.

In the ^1^H NMR spectrum of PSI, the resonance at *δ* 5.27 ppm is due to the methine proton, while the two peaks at *δ* 3.21 and 2.71 ppm correspond to nonequivalent methylene protons. The resonance peaks at *δ* 2.51 and 3.35 ppm correspond to the presence of DMSO-d_6_ and residual water, respectively. Typically, thermally polycondensed PSI shows characteristic signals at *δ* in the range of 6.8–10 ppm, due to the presence of the several irregular structure types in the main chain, like branched and open ring units [44]. The broad peak at *δ* 8.74 ppm is due to the amide protons of branched or ring-open sites. Additionally, the observed signal at *δ* 11.62 ppm is related to the imide proton of the succinimide end group, while the resonance at 5.16 ppm could be attributed to the methine proton of the succinimide end group [45]. The broad peak at *δ* 12.45 ppm indicates the presence of small amounts of the dicarboxylic acid group at the end of the polysuccinimide chain.

The two peaks observed in the ^13^C NMR spectrum at *δ* 173.4 and 172.1 ppm were assigned to two imide (carbonyl) carbons of the succinimide repeating unit, adjacent to the methylene and methine carbons, respectively. Methylene and methine carbons in the main chain appeared in the spectrum at *δ* 32.5 ppm and 47.2 ppm, respectively. Peaks at 48.5 and 33.6 ppm were assigned to the methine and methylene carbons of the succinimide end group, while the two carbonyl carbons in the succinimide end group were observed at 175.7 and 174.4 ppm, respectively [45]. The obtained peak positions for both the ^1^H NMR and ^13^C NMR spectra were similar to the previously reported data for PSI [44,45,46,47] and confirmed that the main polymer chain is composed of a five-membered imide ring. Therefore, the chemical structure of the synthesized PSI was verified by NMR spectroscopy.

In addition, the specific chemical structure of PSI was confirmed by FTIR spectroscopy of the synthesized PSI powder (Table S1). Characteristic absorption bands in FTIR spectra for PSI powder were identified at 1793 cm^−1^ (C-H bending), 1705 cm^−1^ (C=O stretching), 1391 cm^−1^ (C-N stretching), 1258 cm^−1^ (-C-O stretching) and 1163 cm^−1^ (C-C stretching). Thus, FTIR spectroscopy supported the findings obtained by NMR spectroscopy and showed that the final product of the polycondensation reaction is PSI [47].

### 3.2. Morphology of Perovskite Films

The high quality of perovskite films is the key factor for the development of efficient PSCs and stable device operation. The introduction of PSI or PASP polymers possessing large numbers of carbonyl and carboxyl groups, which act as Lewis bases, could passivate defects formed during crystallization at the perovskite grain boundaries and further improve the film morphology.

**Figure 3:**
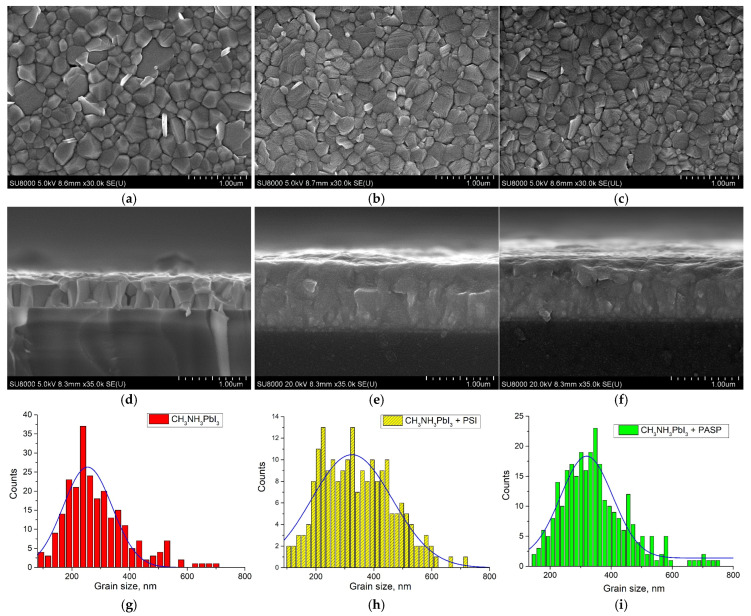
SEM images of the obtained perovskite films: top view (**a**) and cross-section image (**d**) of the pristine perovskite film; top view (**b**) and cross-section image (**e**) of the perovskite film with the addition of 0.1 mg/mL PSI; top view (**c**) and cross-section image (**f**) of the perovskite film with the addition of 0.1 mg/mL PASP. Statistical distribution of perovskite grain sizes for the pristine perovskite film (**g**) and the perovskite films with the addition of PSI (**h**) and PASP (**i**).

Morphological characteristics of pristine and PSI/PASP-modified perovskite films were studied using scanning electron microscopy (SEM). Figure 3 shows the top view and cross-section SEM images for the pristine perovskite film and perovskite films with the PSI or PASP addition. As can be seen from Figure 3a, the pristine perovskite film showed a dense morphology, without visible cracks or large pin-holes. The pristine perovskite material could be characterized by the grain size distribution with an average ± SD value of 255 ± 43 nm (Figure 3g). However, some residual pin-holes and needle-like PbI_2_ crystals can be seen in the SEM images of the pristine perovskite film, as well as some cracking inside the perovskite layer, as is evidenced from the cross-sectional image (Figure 3d).

As can be seen from Figure 3b,c, changes in the morphology of perovskite films could be emphasized upon the addition of the PSI or PASP polymer into the perovskite material. Polymer-modified perovskite film surfaces became denser, without any visible pin-holes and cracks, with denser packing between grains, which could result in a lower number of defects at the grain boundaries. The cross-sectional SEM images for the polymer-modified films further confirm the improved grain packing, showing the absence of grain boundaries along the direction perpendicular to the substrates (Figure 3e,f). PSI-modified perovskite film could be characterized by the broad grain size distribution with an increased average grain size of 326 ± 70 nm (Figure 3h), as compared to the pristine perovskite film. The largest grains have a size of about 700 nm. However, as can be seen from the SEM images, the grains for the PSI-modified perovskite film have a changed orientation with the crystals having a layer-like morphology. As for the PASP-modified perovskite film, its morphological characteristics were similar to those obtained for the PSI-modified perovskite film, with the narrower grain size distribution and an average grain size of 320 ± 42 nm (Figure 3i). It should be noted that the improved morphology for PSI- and PASP-modified perovskite films were observed for quite low (0.1 mg/mL) concentrations of the additives in the perovskite precursor solutions. When higher PSI concentrations were employed (0.5 mg/mL and 3.0 mg/mL), the morphology of perovskite films showed a tendency for deterioration, as was shown by SEM (Figure S1). Therefore, it can be concluded that at higher concentrations (above 0.1 mg/mL), PSI addition begins to impair the morphology, and, consequently, the optoelectronic properties of perovskite films. Similar results were obtained previously for various polymer-modified perovskite films [19,23].

Additionally, the morphology of pristine and PSI/PASP-modified perovskite films was studied using atomic force microscopy (Figure S2). The surface roughness was similar for all perovskite film types, while the average size of the perovskite grains was increased in the order pristine film < PASP-modified perovskite film < PSI-modified perovskite film (Table S2). AFM also showed deterioration in the perovskite film morphology when a high PSI concentration (3.0 mg/mL) was used, manifested in the increased amount of perovskite grains smaller than 100 nm (Figure S3), which is consistent with the results obtained by SEM.

### 3.3. FTIR Spectroscopy of Perovskite Films and Adducts

**Figure 4:**
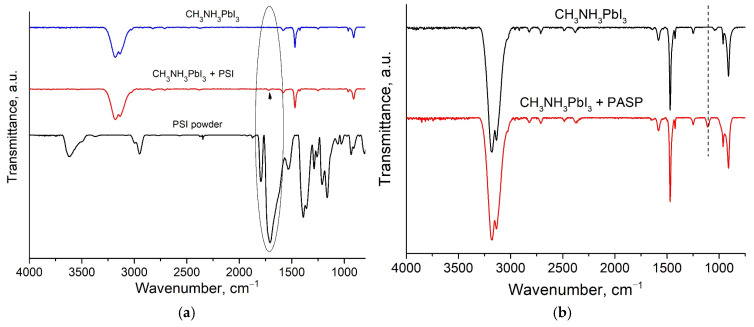

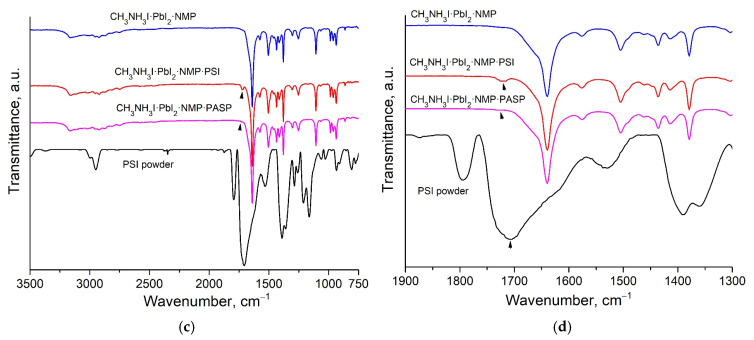
FTIR spectra of PSI powder, pristine perovskite film and perovskite film with added PSI (**a**) and PASP (**b**); FTIR spectra of adduct powders with added PSI and PASP (**c**); and close-up view of the 1900–1300 cm^−1^ region (**d**).

Synthesized adduct powders were studied using Fourier transform infrared spectroscopy to investigate the interactions between perovskite precursors and polymer additives. FTIR measurements were also carried out on perovskite films on Si substrates with or without polymer additives. The FTIR spectra of pristine and polymer-modified perovskite films show that the main peaks are associated with the vibrational modes of the organic cation (Figure 4a,b). The strong peaks between 3300 and 2900 cm^−1^ are due to the -NH stretching vibrations associated with the NH_3_^+^ group of the methylammonium cation [48,49,50].

The remaining peaks could be attributed to the following vibrational modes of CH_3_NH_3_PbI_3_: NH_3_^+^ asymmetric bending (1582 cm^−1^), NH_3_^+^ symmetric bending (1468 cm^−1^), CH_3_ asymmetric bending (1422 cm^−1^), CH_3_-NH_3_^+^ rock (1250 cm^−1^ and 910 cm^−1^) and C-N bond stretch (960 cm^−1^) [49]. Weak vibrational bands at 2958 and 2920 cm^−1^, corresponding to the asymmetric and symmetric CH_3_ stretching [51], were also discernible in the FTIR spectrum of the pristine perovskite film. The main vibrational modes for CH_3_NH_3_PbI_3_ were not shifted upon PSI incorporation into the perovskite film (Figure 4a). The characteristic vibrational bands for the bare PSI powder were observed at 1793 (imide ring), 1705 (C=O bond), 1391 (C-N bond) and 1163 (C-C bond) cm^−1^ (Figure 4a). The addition of polysuccinimide to the perovskite material led to the emergence of the characteristic band at 1720 cm^−1^ related to C=O stretching. Compared to the FTIR spectrum for pure PSI, the C=O stretch vibration peak for the perovskite + PSI film was blue shifted from 1705 to 1720 cm^−1^, which could be evidence of the chemical interaction between the C=O groups in PSI with CH_3_NH_3_^+^ through hydrogen bonding [23] or Pb^2+^ ions through coordination interactions [31]. The observed increase in the vibrational frequency of the C=O bond indicates that the hydrogen bond formation between PSI and perovskite leads to a contraction of the C=O bond length, resulting in a shift to higher wavenumbers in the IR spectrum [52]. Such a phenomenon is often observed in hydrogen-bonded systems, where local partial charge transfer occurs due to solvation or ionic interactions [53]. In addition, the absence of the 1791 cm^−1^ band in the FTIR spectrum of the PSI-modified perovskite film may indicate the partial opening of the imide ring. Similar results were obtained for the perovskite adduct powders upon the addition of PSI (Figure 4c,d), indicating an interaction between the polymer molecules and the perovskite precursors.

The modification of the perovskite film by PASP molecules (Figure 4b) is manifested in the appearance of the characteristic band at 1108 cm^−1^ (C-O stretching) and the disappearance of the band at 1041 cm^−1^ (which belongs to C-H stretching), and an increase in the intensity of the bands in the 3400 cm^−1^ region (-OH groups). The obtained FTIR spectra indicate on the presence of the –COOH groups of polyaspartic acid embedded in the perovskite material. For the FTIR spectrum of the adduct powder with the addition of PASP, the appearance of a band at 1730 cm^−1^, characteristic of C=O bond stretching, is also observed. Summing up the obtained results, FTIR spectroscopy of the perovskite films and adduct powders demonstrated chemical interaction between CH_3_NH_3_^+^ cations and the C=O and COOH groups of PSI and PASP.

Additional experiments on perovskite films were performed using Raman spectroscopy, which also revealed PSI–perovskite interactions, as evidenced by the observed band shifts (Figure S4). Particularly, low frequency Raman bands at around 300–450 cm^−1^ are related to the restricted rotation and torsional modes of CH_3_NH_3_^+^ in CH_3_NH_3_PbI_3_. The appearance of these bands indicate that the organic cation interacts with the inorganic PbI_3_ framework via the NH_3_^+^ through hydrogen bonding [54,55]. The blue shift of this band to higher wavenumbers upon PSI addition indicates the strengthening of the hydrogen bonding, presumably due to the interaction of the cation NH_3_^+^ end with the C=O group of PSI. For the PASP-modified perovskite film, this shift is significantly lower, showing that the interaction of CH_3_NH_3_^+^ cations with the carboxyl groups of PASP is weaker.

### 3.4. XRD

**Figure 5:**
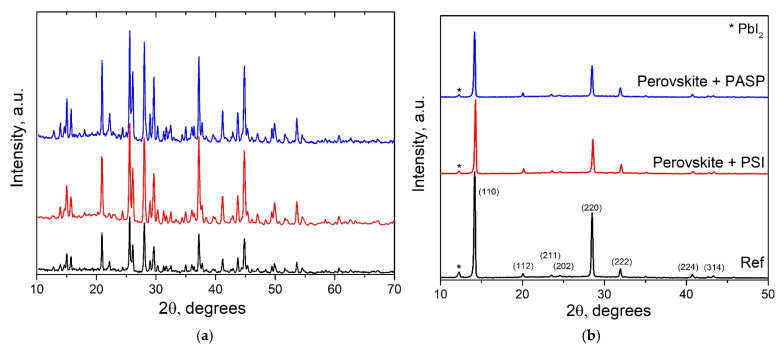
(**a**) X-ray diffraction patterns of adduct powders: CH_3_NH_3_I·PbI_2_·NMP (black), CH_3_NH_3_I·PbI_2_·NMP·PSI (red), CH_3_NH_3_I·PbI_2_·NMP·PASP (blue). (**b**) X-ray diffraction patterns of perovskite films.

To determine how PSI and PASP polymer addition affects perovskite crystal growth, X-ray diffraction (XRD) studies were performed on synthesized adduct powders and perovskite films (Figure 5). In Figure 5a, the XRD patterns of adduct powders (CH_3_NH_3_I·PbI_2_·NMP, CH_3_NH_3_I·PbI_2_·NMP·PSI and CH_3_NH_3_I·PbI_2_·NMP·PASP) show clear and sharp diffraction peaks in the whole 2θ range, which correspond to the formation of the intermediate phases and their molecular ordering [19,20]. No additional peaks were observed in the XRD patterns of adducts with PSI or PASP, indicating that the polymer additives do not change the crystal structure of the intermediate phases. A sharp increase in the intensity of the diffraction peaks for the adducts with PSI and PASP was observed, as compared with the pristine adduct powder, which indicated the enhanced ordering of the intermediate phase induced by polymer addition [23]. The observed significant increase in the intensity of XRD peaks for the adducts with PSI and PASP at high angles (~50° 2θ) could further support the improved long-range ordering of the intermediate phase due to PSI or PASP addition.

After crystallization, most of the peaks corresponding to the intermediate phases disappeared with the formation of the typical CH_3_NH_3_PbI_3_ perovskite diffraction pattern. It is interesting to note that the intensity of the main XRD peaks for the polymer-modified perovskite films did not increase. On the other hand, the intensity ratio between the main two diffraction peaks, namely, (110) and (220), clearly changed for the polymer-modified perovskite films, as compared to the pristine one. This could be evidence for the decreased film orientation upon addition of PSI or PASP into the perovskite layer, which is anticipated as multiple grains differently oriented in the vertical direction [56]. This suggestion is supported by the observed changes in the grain size and film morphology upon PSI or PASP addition, as can be seen from the SEM images (Figure 3). Furthermore, the XRD peak related to PbI_2_ at 12.3° 2θ was significantly reduced for both polymer-modified perovskite film samples. Since excess PbI_2_ could act as defects and recombination centers in the perovskite films [31], the decrease in PbI_2_ content due to PSI/PASP modification could lead to reduced defect density at the grain boundaries and increased photovoltaic performance.

### 3.5. Optical Properties of Perovskite Films

As was shown above, the presence of the PSI and PASP polymers in the perovskite films was confirmed by FTIR and the polymer–perovskite interactions were evidenced in the adduct powders and films, which could lead to the passivation of defects at the grain boundaries [9,19]. To elucidate the effects of these interactions, the optoelectronic properties of the films were studied by UV-Vis and photoluminescence (PL) spectroscopy.

**Figure 6:**
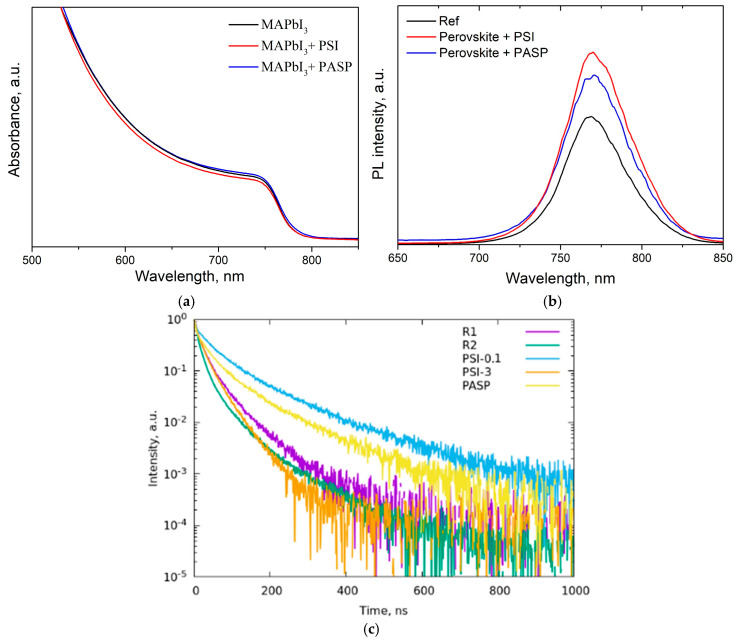
(**a**) UV-Vis absorption spectra for pristine and polymer-added perovskite films on glass substrates. (**b**) Steady-state PL spectra of pristine and polymer-added perovskite films. (**c**) Time-resolved photoluminescence (TRPL) spectra for pristine perovskite films (R1 and R2), perovskite films with various levels of PSI addition (PSI-0.1 and PSI-3) and perovskite film with the addition of 0.1 mg/mL of PASP.

The UV-VIS spectra of perovskite films (Figure 6a) do not show significant changes in terms of bandgap and absorbance upon the addition of PSI or PASP into the perovskite film. This was confirmed by the steady-state photoluminescence (PL) spectra (Figure 6b), which did not show a significant shift in the PL peak positions for the studied perovskite films. However, both PSI and PASP addition leads to the significantly increased intensity of the PL peak, as compared to pristine perovskite. This could be evidence of the increase in the lifetime of photogenerated charge carriers in the perovskite film as a result of polymer addition. Time-resolved photoluminescence (TRPL) was measured to probe charge carrier behavior in the perovskite films (Figure 6c). As can be seen, the pristine perovskite film showed the fastest TRPL decay, while the addition of PSI and PASP significantly enhanced the PL lifetime. The PSI-modified perovskite film with 3.0 mg/mL PSI in the perovskite precursor showed the fastest PL decay, indicating that the deterioration of the perovskite film morphology shown by SEM (Figure S1) can lead to a decrease in the lifetime of photogenerated charge carriers.

Time-resolved PL curves were fitted using a 4-exponential decay model, f(t) = A_1_ exp(−(t − t0)/τ_1_) + A_2_ * exp(−(t − t0)/τ_2_) + A_3_ * exp(−(t − t0)/τ_3_) + A_4_ * exp(−(t − t0)/τ_4_). In this model, the faster decay components (τ_1_ and τ_2_) are attributed to charge carrier trapping involving defects, and slower decay components (τ_3_ and τ_4_) are assigned to the bimolecular radiative recombination in the bulk of the perovskite layer [23,57]. The obtained fitted parameters according to the model used are presented in Table 1.

PSI or PASP addition leads to a twofold increase in the time constant for the fast decay component τ_2_ (34.6 and 28.6 ns, respectively) as compared to the bare perovskite film (16.6 ns), which indicates the reduced defect density in the modified perovskite layers as a result of defect passivation by the introduced polymers [23,26,31]. The slower decay component τ_3_ was also significantly enhanced from 61.6 ns (bare perovskite) to 96.7 ns (PSI) and 87.3 ns (PASP), suggesting that PSI or PASP addition reduced recombination losses in the perovskite bulk, which could be attributed to the enhanced film morphology and increased grain sizes [23,29]. PSI was shown to be superior in increasing the charge carrier lifetime in perovskite films as compared to PASP due to more uniform film morphology for the PSI-added perovskite film, as evidenced by SEM (Figure 3). Therefore, the introduction of PSI or PASP into the perovskite film simultaneously decreased the perovskite crystal defects and suppressed recombination losses, giving rise to enhanced charge carrier dynamics and possible improvement in the photovoltaic performance.

### 3.6. Photovoltaic Performance of PSCs

**Figure 7:**
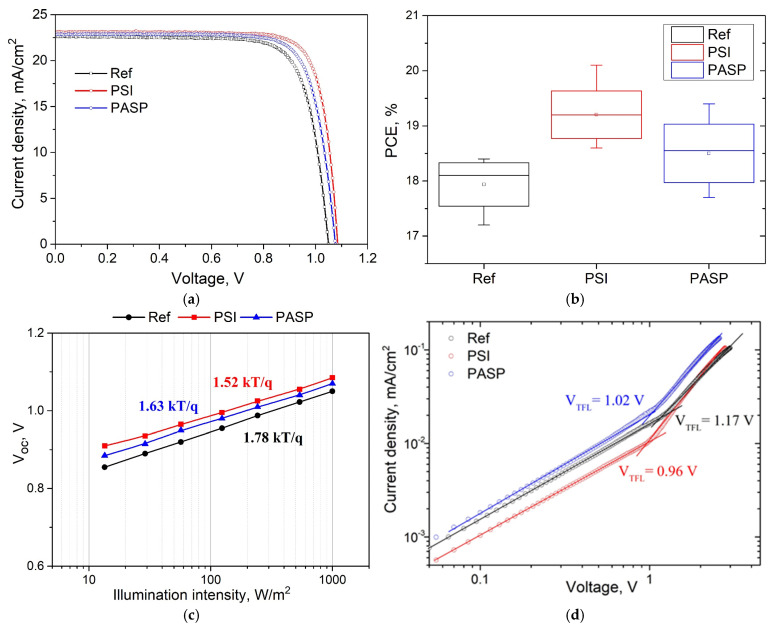
(**a**) J-V curves of the perovskite solar cells with and without PSI and PASP (optimized concentration of 0.1 mg/mL in the precursor solution), (**b**) statistical distribution of PCE for studied PSCs (10 PSC samples for each series), (**c**) light intensity-dependent measurements of V_OC_ and (**d**) dark I-V curves of hole-only devices (glass/FTO/PEDOT:PSS/perovskite/spiro-MeOTAD/Au).

Since the photovoltaic properties of PSCs largely depend on the number of defects inside the perovskite film and at the interfaces, the introduction of a polymer material with carbonyl and carboxyl groups can passivate defects formed during perovskite crystallization and, therefore, improve the photovoltaic performance of PSCs [23,31]. The photovoltaic characteristics of perovskite solar cells with different polymer additives were compared, as shown in Figure 7. The devices had a planar glass/FTO/TiO_2_/SnO_2_/perovskite/spiro-MeOTAD/Au architecture.

J-V curves for the best performing devices are shown in Figure 7a and for the champion PSC with PSI addition, a PCE value of 20.1% was obtained (Table 2). It should be noted that quite low concentrations of PSI and PASP in the perovskite precursor solutions (0.1 mg/mL in the perovskite precursor solution) was found to be optimal for perovskite modification and enhancement of the PV characteristics. When higher PSI concentrations (0.5 and 3.0 mg/mL) were added to the precursor solutions, the photovoltaic properties of the respective PSCs were drastically decreased (Figure S5), presumably due to the observed deterioration of the perovskite film morphology, as was shown by SEM (Figure S1). Therefore, all further photovoltaic characteristics refer to the PSCs with 0.1 mg/mL polymer concentration in the precursor solution.

Both perovskite modifications using PSI and PASP resulted in increased average PCE values for the respective PSCs up to 19.1% and 18.3%, as compared to the reference PSCs showing a 17.9% average PCE value (Table 2). Average V_OC_ and FF values were increased as well both for PSI- and PASP-modified PSCs. In addition, higher EQE values were observed for both polymer-modified PSCs compared to the reference device (Figure S6). As can be seen, PSI modification provided better enhancement of the PV parameters of PSCs, which correlates with the TRPL results described above.

The observed increase in the photovoltaic parameters (Voc and FF) could be attributed to the decrease in the density of defects in the perovskite film. This was furthermore demonstrated by the V_OC_ vs. light intensity plots, which gave reduced slope values for PSI-modified (1.52 kT/q) and PASP-modified (1.63 kT/q) PSCs, as compared to the reference device (1.78 kT/q). When the slope value (ideality factor) is close to 2, trap-induced Shockley−Reed−Hall (SRH) recombination is the dominant process in the perovskite layer, while slope values close to 1 indicate predominant bimolecular recombination. The observed decrease in the ideality factor values confirms efficient suppression of trap-assisted recombination provided by PSI and PASP modification due to effective defect passivation [29,58], which is in agreement with the TRPL results. Similar trends were observed from the dark I-V curves for hole-only devices, which show decreases in the V_TFL_ values as a result of PSI and PASP modification. These findings also confirm that PSI and PASP addition could efficiently passivate defects in the perovskite layer.

**Figure 8:**
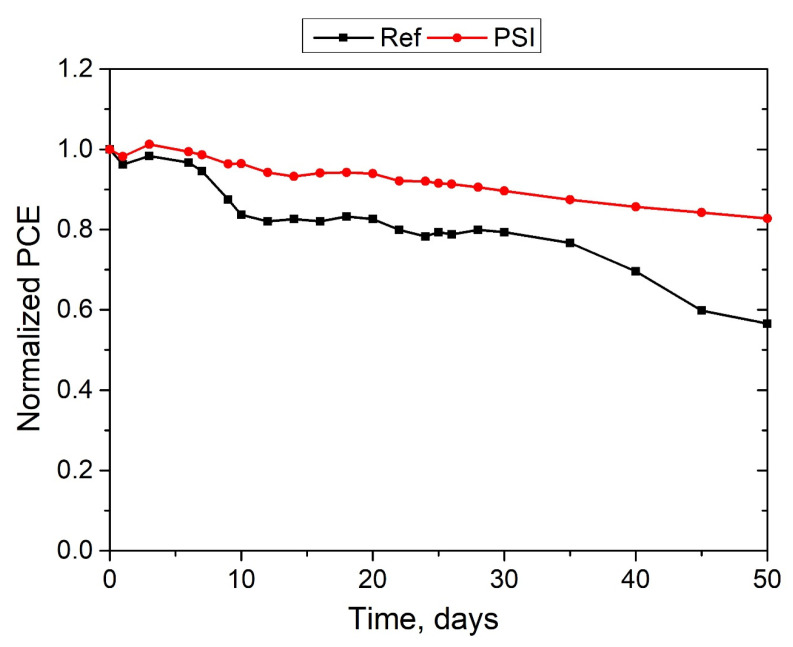
Shelf-stability of unencapsulated perovskite solar cells under ambient conditions (normalized PCE values).

Granted that a large number of defects in the perovskite films is detrimental to PSC stability, we studied the evolution of PCE for PSI-modified PSCs during prolonged storage under ambient conditions. The long-term stability of PSCs was evaluated in ambient conditions with reduced humidity (30%) without encapsulation. Figure 8 illustrates the changes in the normalized PCE values for the studied PSCs.

As can be seen, after 40 days of storage, the PSI-modified device retained 83% of its original power conversion efficiency (PCE), whereas the pristine PSC device showed an almost 45% reduction in its initial PCE. This indicates that PSI modification of perovskite films could provide significant improvement in PSC stability.

## 4. Conclusions

In this study, the modification of perovskite films with low-cost green polymers (PSI and PASP) was investigated. Structural, morphological and optoelectronic properties of polymer-modified perovskite films were scrutinized by FTIR and UV-Vis spectroscopy, scanning electron microscopy (SEM) and X-ray diffraction (XRD). FTIR results evidence the interaction of PSI with the methylammonium cation and/or Pb^2+^ ions in the perovskite adducts and films, which is evidenced by the blue shift to 1720 cm^−1^ for the PSI C=O stretching band. Polymer-modified perovskite films showed improved grain packing and increased average grain size, as compared to the pristine perovskite film. SEM and XRD results indicate that the addition of PSI provides a decrease in the excess PbI_2_ content at the surface of the perovskite grains, inhibiting the formation of defects. Time-resolved photoluminescence (TRPL) showed that the average lifetime of charge carriers was significantly increased after the introduction of PSI and PASP polymers into the perovskite film, with PSI being more effective as compared to PASP in this respect. As a result, the observed changes in the structural and optoelectronic properties of perovskite films led to enhancements in the PCE values of up to 20% for planar n-i-p perovskite solar cells based on PSI-modified perovskite layers. The primary factor contributing to the improved performance of PSI-modified perovskite solar cells was the reduction in the recombination losses and increase in charge carrier lifetime, attributed to the effective passivation of surface defects. Additionally, PSI-modified PSCs showed enhanced stability, retaining 80% of their initial efficiency after storage of the unencapsulated devices for 40 days under ambient conditions. These findings provide a promising approach for the efficient application of low-cost, green polymer materials in perovskite photovoltaics, opening the way for the improved performance and long-term stability of perovskite solar cells. The proposed perovskite modification strategy could be further extended for altered perovskite formulations, including formamidinium-based perovskites. Moreover, other types of poly(amino acids) with suitable functional groups could be considered as additives for perovskite materials.

## Figures and Tables

**Table 1:** Fitted parameters of the experimental TRPL profiles for the studied perovskite film samples using the 4-exponential decay model.

	Ref	PSI	PASP
A_1_	414,995	542,168	311,550
A_2_	256,565	772,937	249,959
A_3_	339,670	506,740	116,449
A_4_	8746	61,970	12,386
τ_1_, ns	4.0	3.9	3.2
τ_2_, ns	16.6	34.6	28.6
τ_3_, ns	61.6	96.7	87.3
τ_4_, ns	210.2	244.6	217.8

**Table 2:** The average PV parameters of pristine PSCs and PSCs with PSI and PASP addition.

Sample	J_SC_, (mA/cm^2^)	V_OC_, (V)	FF	PCE, (%)
Reference PSC	22.43 ± 0.48 ^a^ (22.45)	1.050 ± 0.01 ^a^ (1.045)	0.765 ± 0.014 ^a^ (0.78)	17.9 ± 0.4 ^a^ (18.3)
PSC with PSI (0.1 mg/mL)	22.55 ± 0.37 ^a^ (23.1)	1.077 ± 0.005 ^b^ (1.085)	0.791 ± 0.012 ^b^ (0.803)	19.10 ± 0.25 ^b^ (20.1)
PSC with PASP (0.1 mg/mL)	22.16 ± 0.39 ^a^ (22.82)	1.076 ± 0.009 ^b^ (1.078)	0.779 ± 0.017 ^b^ (0.785)	18.5 ± 0.4 ^c^ (19.4)

Values represent mean ± SD. Values for the champion cell of each PSC type are given in parentheses. Values within each column with different superscripts are statistically different at 0.05 significance level.

## Data Availability

The original contributions presented in this study are included in the article/Supplementary Materials. Further inquiries can be directed to the corresponding author.

## Supplementary Materials

The following supporting information can be downloaded at: https://www.mdpi.com/article/10.3390/polym17050653/s1, Table S1. Characteristic bands in FTIR spectra for PSI and PASP powders, Perovskite + PSI and Perovskite + PASP films; Table S2. Root mean square roughness *S_q_* and average particle size *D* for studied perovskite films obtained from AFM images; Figure S1. Top view SEM images of perovskite films obtained using perovskite precursor solutions with increased PSI concentration of 0.5 mg/mL (a) and 3.0 mg/mL (b); Figure S2. AFM images of pristine perovskite film (a) and perovskite films obtained with the addition of PSI (b) and PASP (c). Polymers were added to the perovskite precursor solutions at concentration of 0.1 mg/mL; Figure S3. AFM image of perovskite film obtained using perovskite precursor solution with 3.0 mg/mL PSI (a) and the corresponding particle size distribution (b). The average particle size was obtained as 58 ± 10 nm; Figure S4. Raman spectra of pristine perovskite film and perovskite films obtained with the addition of PSI and PASP. Spectra were obtained using excitation wavelength of 532 nm. Band positions of the corresponding Raman bands are indicated; Figure S5. J-V curves for PSCs obtained using perovskite precursor solutions with increased PSI concentration (0.5 mg/mL and 3.0 mg/mL); Figure S6. EQE spectra for the PSCs with and without PSI and PASP. Optimized polymer concentration of 0.1 mg/mL in the perovskite precursor solution was used.
