# Supplementary material for: Defect Passivation in Perovskite Solar Cells Using Polysuccinimide-Based Green Polymer Additives
Source: Polymers (Basel). 2025 Feb 28;17(5):653. doi: 10.3390/polym17050653 (PMC11902386; doi:10.3390/polym17050653)
Supplement: Supplementary file 1 [file polymers-17-00653-s001.zip › polymers-3472351-supplementary.pdf]

Supplementary materials for:

Defect passivation in perovskite solar cells using polysuccinimide-based green polymer additives

Olga V. Alexeeva[1*], Anna B. Nikolskaia[1*], Vasilisa I. Petrova[1], Olga K. Karyagina[1], Alexey L. Iordanskii[2], Liudmila L. Larina[1], Oleg I. Shevaleevskiy[1]

[1] Emanuel Institute of Biochemical Physics, Russian Academy of Sciences, 119334 Moscow, Russia
[2] N.N. Semenov Federal Research Center for Chemical Physics Russian Academy of Sciences, 119991 Moscow, Russia
Correspondence: O.A., alexol@yandex.ru;

Table S1. Characteristic bands in FTIR spectra for PSI and PASP powders, Perovskite + PSI and Perovskite + PASP films.

Perovskite characteristic bands, cm^{-1}	PSI characteristic bands, cm^{-1}	Perovskite + PSI characteristic bands, cm^{-1}	PASP characteristic bands, cm^{-1}	Perovskite + PASP characteristic bands, cm^{-1}	Characteristic band assignment
-	3616	-	3411	3411	-OH
3175	3361	3175	3392	3180	$-NH_3^+$ (asim)
3130		3130		3130	$-NH_3^+$ (sim)
2975	2993	2975		2925	-CH (asim)
2932	2950	2932		2910	-CH (sim)
2820	-	2820	-	2820	$-NH_3^+$ - CH_3 - NH_3^+
2711	-	2711	-	-	$-NH_3^+$ (bending asim+sym)
-	1793	-	-	-	C-H (bending sym)
-	1705	1716	1716	1730	C=O (stretching)
-	-	-	1646	1646	C-C stretching
1582	-	1582	1560	1583	$-NH_3^+$ (bending asim)
1468	-	1468	-	1475	$-NH_3^+$ (bending sim)
1422	-	1422	-	1420	$-CH_3$ (asim bending)
-	1391	1393	1393	1393	C-N (stretching)
-	1258	1250	1190	-	-C-O (stretching)
-	1163	-	-	-	C-C (stretching)
-	1041	1027	1108	1108	C-O (stretching)
960	-	960	-	960	C-N stretching
910	-	910	-	910	CH_3-NH_3^+ (rocking)

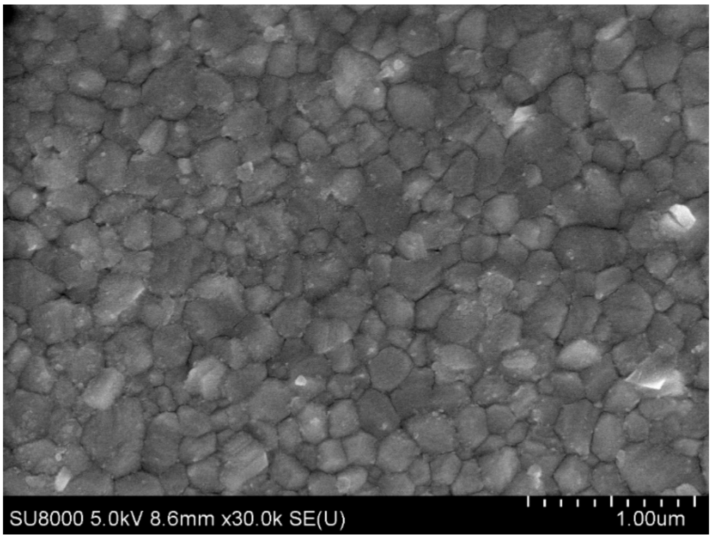

(a)

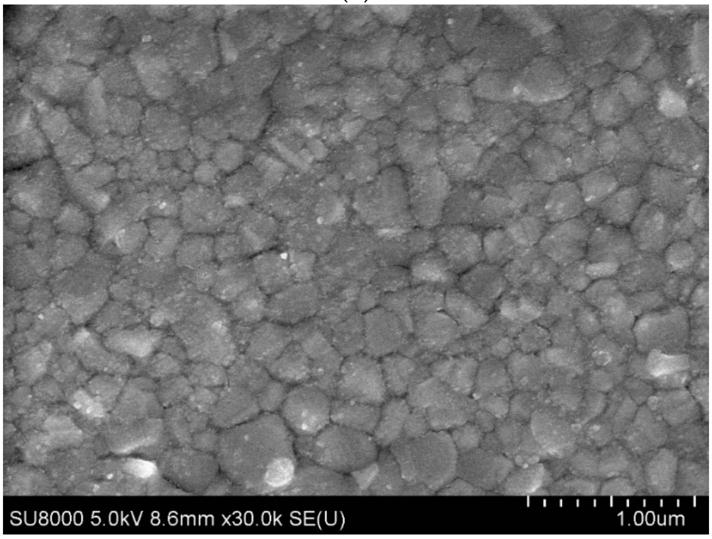

(b)

Figure S1. Top view SEM images of perovskite films obtained using perovskite precursor solutions with increased PSI concentration of 0.5 mg/mL (a) and 3.0 mg/mL (b).

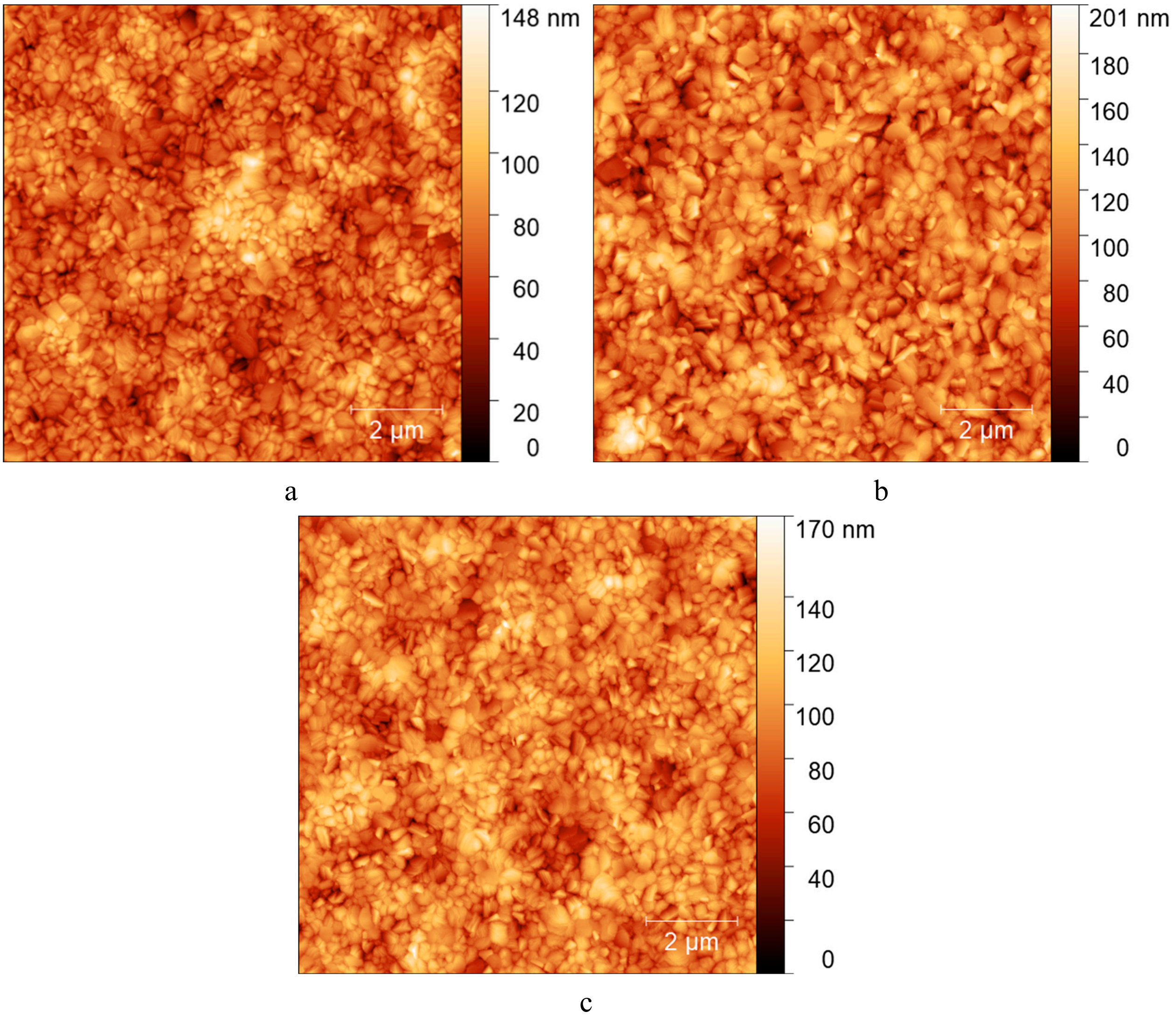

Figure S2. AFM images of pristine perovskite film (a) and perovskite films obtained with the addition of PSI (b) and PASP (c). Polymers were added to the perovskite precursor solutions at concentration of 0.1 mg/mL.

Table S2. Root mean square roughness S_q and average particle size D for studied perovskite films obtained from AFM images.

Sample	S_q, нм	D, нм
Pristine $CH_3NH_3PbI_3$	18 ± 8	183 ± 3
$CH_3NH_3PbI_3$ + PSI 0.1 mg/mL	24 ± 4	238 ± 5
$CH_3NH_3PbI_3$ + PASP 0.1 mg/mL	17.4 ± 1.0	205 ± 2

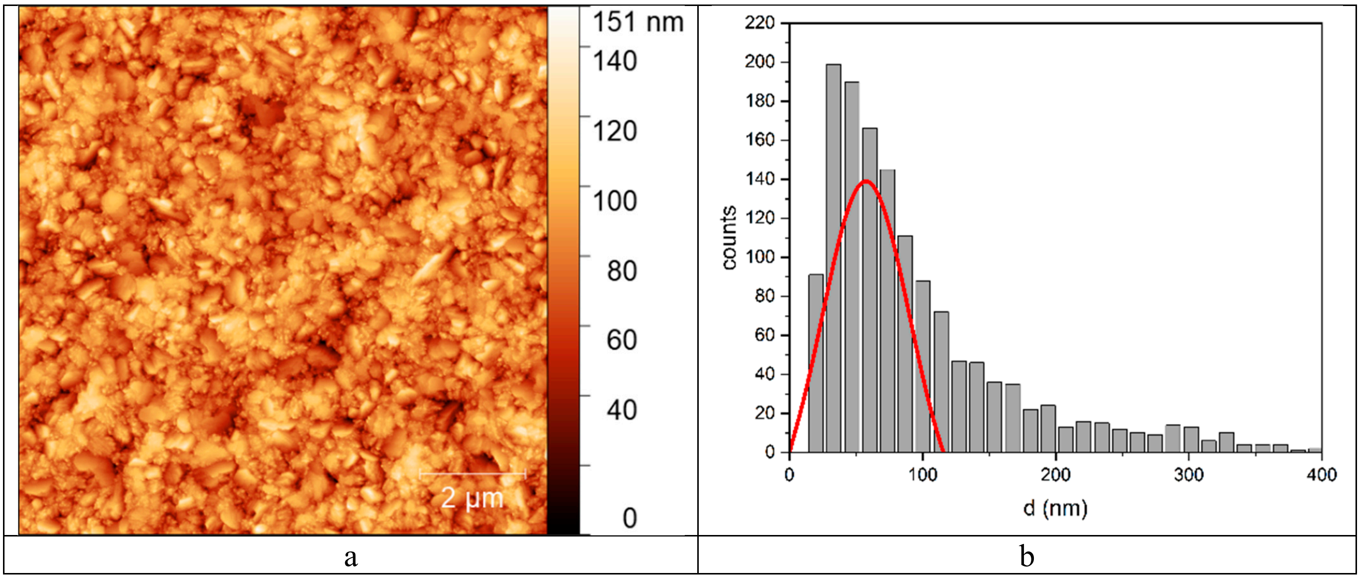

Figure S3. AFM image of perovskite film obtained using perovskite precursor solution with 3.0 mg/mL PSI (a) and the corresponding particle size distribution (b). The average particle size was obtained as 58 ± 10 nm.

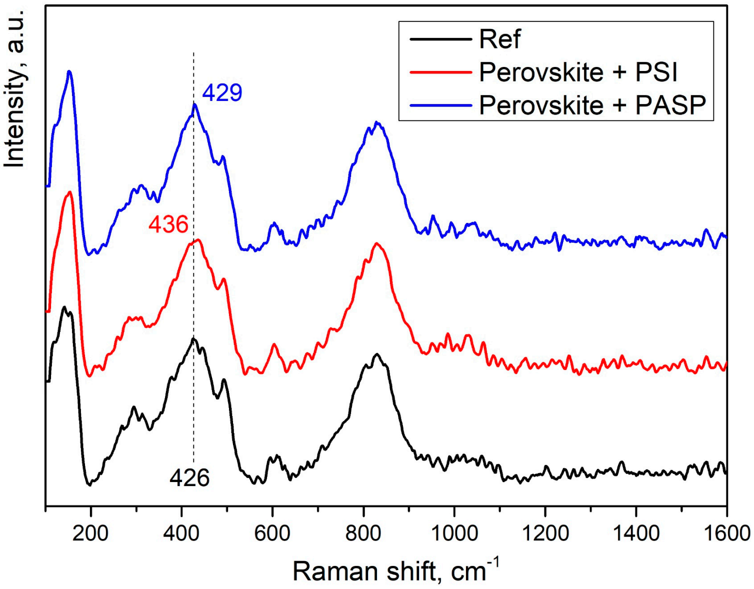

Figure S4. Raman spectra of pristine perovskite film and perovskite films obtained with the addition of PSI and PASP. Spectra were obtained using excitation wavelength of 532 nm. Band positions of the corresponding Raman bands are indicated.

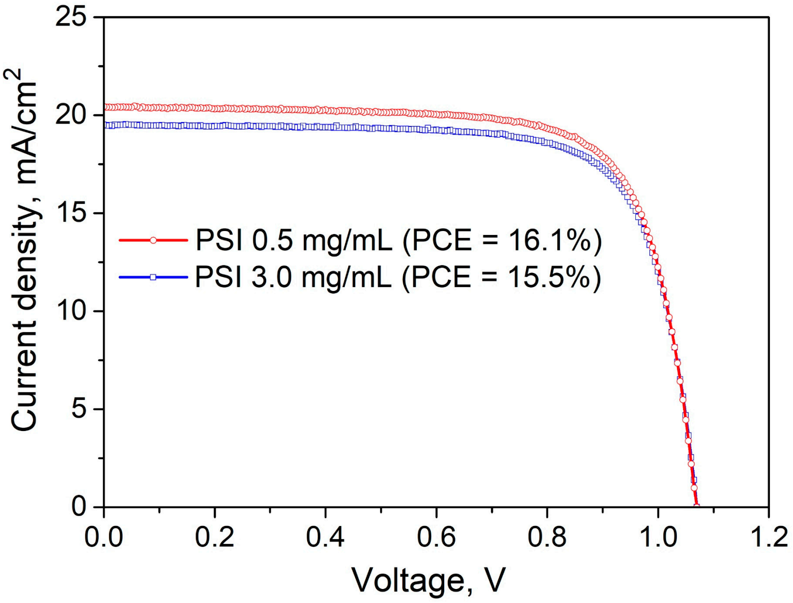

Figure S5. J-V curves for PSCs obtained using perovskite precursor solutions with increased PSI concentration (0.5 mg/mL and 3.0 mg/mL).

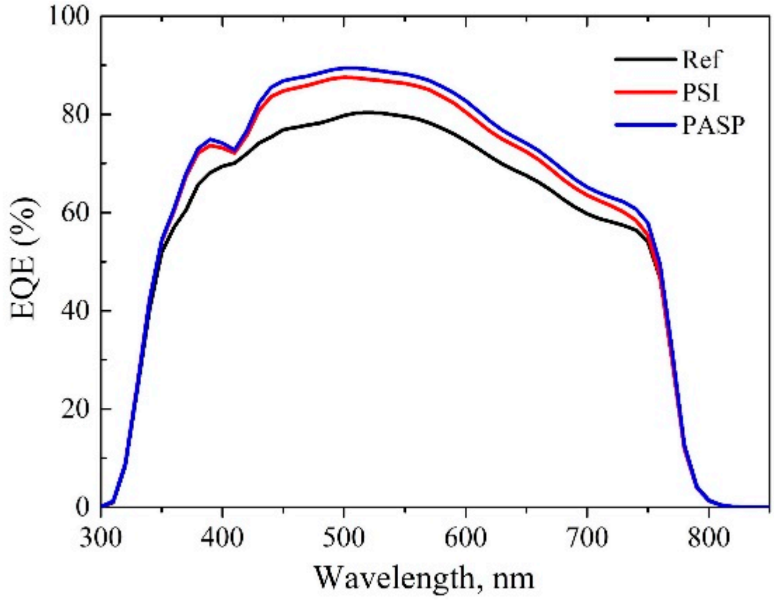

Figure S6. EQE spectra for the PSCs with and without PSI and PASP. Optimized polymer concentration of 0.1 mg/mL in the perovskite precursor solution was used.
